# The Diseases and Deformities of the Fœtus

**Published:** 1896-03

**Authors:** 


**The Diseases and Deformities of the Fœtus.** By J. W. Ballantyne, M.D. Vol. II. Pp. xi., 264. Edinburgh: Oliver and Boyd. 1895.

This the second volume of Dr. Ballantyne's admirable work on ante-natal pathology is devoted to congenital diseases of the subcutaneous tissues and skin. The diseases chiefly considered are sclerema neonatorum and ichthyosis. The references to the literature of the various forms of skin disease are very abundant, and the work is, as all Dr. Ballantyne's books are, evidently the result of much laborious study, and is a very valuable guide to the subject of which it treats.

